# The impact of ZTE-based MR attenuation correction compared to CT-AC in ^18^F-FBPA PET before boron neutron capture therapy

**DOI:** 10.1038/s41598-024-63248-9

**Published:** 2024-06-17

**Authors:** Yi-Wen Lo, Ko-Han Lin, Chien-Ying Lee, Chia-Wei Li, Chien-Yuan Lin, Yi-Wei Chen, Ling-Wei Wang, Yuan-Hung Wu, Wen-sheng Huang

**Affiliations:** 1 Integrated PET/MR Imaging Center, Department of Nuclear Medicine, Taipei Veterans General Hospital, Taipei, Taiwan ROC; 2 Department of Biomedical Imaging and Radiological Sciences, National Yang-Ming Chiao Tung University, Taipei, Taiwan ROC; 3 GE Healthcare, Taipei, Taiwan ROC; 4 Department of Nuclear Medicine, Chang Bing Show Chwan Memorial Hospital, Taipei, Taiwan ROC; 5 Division of Radiotherapy, Department of Oncology, Taipei Veterans General Hospital, Taipei, Taiwan ROC; 6 Clinical Imaging Research Center (CIRC), Yong Loo Lin School of Medicine, National University of Singapore, Singapore, Singapore

**Keywords:** PET/MR, PET/CT, ZTE, Attenuation correction, BNCT, FBPA, Cancer, Biomarkers, Health care, Medical research, Molecular medicine, Oncology

## Abstract

Tumor-to-normal ratio (T/N) measurement of ^18^F-FBPA is crucial for patient eligibility to receive boron neutron capture therapy. This study aims to compare the difference in standard uptake value ratios on brain tumors and normal brains using PET/MR ZTE and atlas-based attenuation correction with the current standard PET/CT attenuation correction. Regarding the normal brain uptake, the difference was not significant between PET/CT and PET/MR attenuation correction methods. The T/N ratio of PET/CT-AC, PET/MR ZTE-AC and PET/MR AB-AC were 2.34 ± 0.95, 2.29 ± 0.88, and 2.19 ± 0.80, respectively. The T/N ratio comparison showed no significance using PET/CT-AC and PET/MR ZTE-AC. As for the PET/MRI AB-AC, significantly lower T/N ratio was observed (− 5.18 ± 9.52%; *p* < 0.05). The T/N difference between ZTE-AC and AB-AC was also significant (4.71 ± 5.80%; *p* < 0.01). Our findings suggested PET/MRI imaging using ZTE-AC provided superior quantification on ^18^F-FBPA-PET compared to atlas-based AC. Using ZTE-AC on ^18^F-FBPA-PET /MRI might be crucial for BNCT pre-treatment planning.

## Introduction

Boron Neutron Capture Therapy (BNCT) has become a promising cancer treatment scheme that uses the neutron capture and fission reaction of the ^10^B boron isotope (^10^B). The fission reaction is induced by an epithermal neutron and yields a α particle with high linear energy transfer (LET) and a ^7^Li particle^1^. To achieve an expected treatment response and to mitigate the adverse effect, BNCT requires precise estimation of ^10^B concentration not only in the target tumor but in neighboring normal tissues^2^. Presently, the theranostic combination of the ^10^B boron-carrier “boronophenylalanine (BPA)” and the imaging probe [^18^F]-fluoro-BPA ([^18^F]-FBPA) have been used in most contemporary clinical treatments. The FBPA image by PET/CT scanner is implemented for treatment plans and uptake measurements^3^ and is the modality of choice to monitor the ^10^B boron-carrier distribution before BNCT treatment.

To ensure sophisticated treatment plans for BNCT, physicians need to determine precisely the ^10^B concentrations in targeted lesions and the surrounding normal tissues^4^ with a tumor-to-normal ratio(T/N ratio) ≥ “2.5”^5–9^. However, the CT in PET/CT provides brain anatomical images inferior to those of the magnetic resonance (MR) technique in PET/MR; therefore, the limited spatial resolution from PET/CT increases the difficulty in tumor delineation for precision BNCT planning, especially in brain regions^10^. Consequently, PET/MR has become more commonly used in the era of contemporary molecular imaging because it provides comprehensive anatomical information without the ionizing radiation exposure that occurs with CT. The simultaneous morphological and metabolic imaging of PET/MR fulfilled the requirements for intricate BNCT planning in brain tumors, especially for tumor boundary recognition and segmentation. Moreover, PET/MR is also promising for normal tissue determination since a previous report found that the delineation of normal tissue significantly influenced the T/N ratio measurement and treatment management.

Alternatively, to measure the ^18^F-FBPA standardized uptake value (SUV) and SUV ratio (SUVR) in PET/MR, it is necessary to perform photon-attenuation correction (AC) for optimized photon quantification. Photon attenuation in the body is mainly caused by soft tissues, bone and MRI hardware components^11–13^. In practice, this attenuation remains even with consistent agreement between SUVRs obtained from PET/CT and PET/MR. However, PET/MR AC remains debatable^14^. Existing PET/CT and PET/MR data for [^18^F]-fluorodeoxyglucose ([^18^F]-FDG) PET imaging indicated that MR-based AC is accurate and reliable in most tissue types, with SUVR deviations generally less than 10%^15^. With the development of hybrid modalities, there are four different AC approaches available in clinical use: PET/CT AC (CT-AC), atlas-based AC (AB-AC), ultrashort echo-time AC (UTE-AC), and zero echo-time AC (ZTE-AC). Because the image principle of segmentation-based AC will cause a considerable loss of bone signals^16^, especially in postoperative patients with deformed skull^17^, an alternative AC scheme for better clinical applications is required. With the advent of the ZTE technique, PET/MR AC has been advocated to overcome limitations for whole-body^18^ and whole brain^19^ studies. Although a slight underestimation might occur in the whole-brain fields^17,20, 21^, existing data indicates that ZTE-AC has been proven to offer various benefits for radiotherapy planning in the brain^16,19^. Previous FDG studies also have found that ZTE-AC provided an advanced quantitative evaluation in brain PET^18,21, 22^. However, to our knowledge, no report has focused on ^18^F-FBPA brain PET/MR yet. Thus, this study aimed to evaluate and compare the effects of ZTE-AC and AB-AC on the power of PET/MR quantification with those of the standard CT-AC in normal-brain and tumor-brain tissues.

## Methods

### Participants and tumor VOI (volume of interest) selection

Between July 2019 to January 2022, a total of 28 patients with confirmed brain tumors (17 females and 11 males, mean age = 56 ± 15.97 years old) who were potential candidates for BNCT were enrolled (Table 1). All received PET/CT and PET/MR examinations were performed on the same day. The patients’ tumor types included 14 glioblastomas, 3 atypical meningiomas, 2 astrocytomas, 3 anaplastic astrocytomas, 1 oligoastrocytoma, 3 anaplastic oligodendrogliomas, and 3 metastatic cancers. The tumor regions of all datasets were delineated manually by an experienced radiographer and double-checked by an experienced nuclear medicine physician. This study was approved by the Institutional Review Board of Taipei Veterans General Hospital (IRB: 2022-11-003AC).

**Table 1:** Summary of patients’ demography.

Number of enrolled patients	28
Patients’ age (year-old)	56 ± 15.97 (19–88)
Male/female	11/17
Mean PET/CT scanning time (min)	34.2 ± 2.3 (30–44)
FBPA activity at the start of PET/CT(MBq)	159 ± 34.9 (109–260)
Mean PET/MRI scanning time (min)	68.4 ± 4.6 (63–82)
FBPA activity at the start of PET/MRI (MBq)	128 ± 26.8 (86–188)

Type of brain tumors	
GBM	14
Atypical meningioma	3
Astrocytoma	2
Anaplastic astrocytoma	3
Oligoastrocytoma	1
Anaplastic oligoastrocytoma	2
Metastatic cancer	3

*FBPA* ^18^F-labeled boronophenylalanine, *GBM* glioblastoma, *MBq* million Becquerel.

### Data acquisition

Data were acquired on a PET/CT scanner (Discovery MI DR; GE Healthcare, Milwaukee, WI, USA) and a 3T PET/MR scanner (SIGNA PET/MR; GE Healthcare, Milwaukee, WI, USA) using a geometry-embracing method (GEM) head-and-neck unit for signal detection and a whole-body coil for radio-frequency excitation. The following settings were used to acquire the helical CT scans in PET/CT: 120 kVp; 30–130 mA; slice thickness of 3.75 mm; field of view (FOV) 512 × 512. In PET/MR imaging, some clinical images were acquired for diagnostic purposes before PET scan, including fast-SE (FSE) with inversion-recovery axial plane T1-weighted imaging with a repetition time (TR) of 1800 ms, echo time (TE) of 23 ms, inversion time (TI) of 750 ms), FSE coronal plane T2-weighted imaging (TR, 4545 ms; TE, 110 ms), and T2-fluid-attenuated inversion recovery imaging (TR, 9000 ms; TE, 92 ms; TI, 2472 ms). Single echo-planar-imaging based diffusion-weighted images (TR, 6000 ms; TE, 77 ms) were also acquired with a *b*-value of 1000 s/mm^2^. Then, the ZTE sequence (TR/TE = 4.048/0.0167 ms; FOV: 60 × 60 cm; matrix size: 128 × 128; slice thickness: 2.78 mm; flip angle: 5°) was used for ZTE-AC, with one structural imaging dataset, liver imaging with volume acceleration flexible (LAVA Flex), and the Dixon method. The LAVA Flex parameters were TR/TE of 5.35/1.936 ms, FOV of 40 × 40 cm, matrix size of 512 × 512, slice thickness of 2 mm, and flip angle of 12°. The Dixon method parameters were TR/TE of 4/1.1 and 2.2 ms, FOV of 50 × 37.5 cm, matrix size of 256 × 128; slice thickness of 5.2 mm, and flip angle of 5° (Table 2).

**Table 2:** Imaging parameters for PET/MRI and PET/CT.

	LAVA-Flex	Dixon method	Zeo echo time	CT-AC
Repetition time (ms)	5.35	4	4.048	–
Echo time (ms)	1.936	1.1 & 2.2	0.0167	–
Flip angle (°)	12	5	5	–
Field of view (mm)	40 × 40	50 × 37.5	60 × 60	–
Matrix size	512 × 512	256 × 128	128 × 128	512 × 512
Slice thickness (mm)	2	5.2	2.78	3.75
Kilovoltage peak, kVp	–	–	–	120
Milliampere-seconds, mAs	–	–	–	30–130

*LAVA* liver acceleration volume acquisition, *CT-AC* attenuation correction of computed tomography.

The PET/CT and PET/MR scans were acquired at 34.2 ± 2.3 min and 68.4 ± 4.6 min after intravenous injection, respectively. In both modalities, PET data were accumulated for 15 min to reconstruct the ^18^F-FBPA PET images. The average radioactivity at the start of the ^18^F-FBPA PET scanning was 159 ± 34.9 MBq in PET/CT (dose range: 109–260 MBq) and 128 ± 26.8 MBq in PET/MR (dose range: 86 -188 MBq) as shown in Table 1. In PET/MRI, two AC maps were generated by the ZTE-AC and AB-AC methods. The reconstruction algorithm was used with the following settings: 2 iterations, 28 subsets, 5-mm filter cutoff, 192 × 192 matrix size, and 60-cm display field-of-view (DFOV). In PET/CT, the reconstruction algorithm was used with 2 iterations, 24 subsets, 5-mm filter cutoff, 256 × 256-matrix size, and 70-cm DFOV.

### Data preprocess and analysis

**Figure 1:**
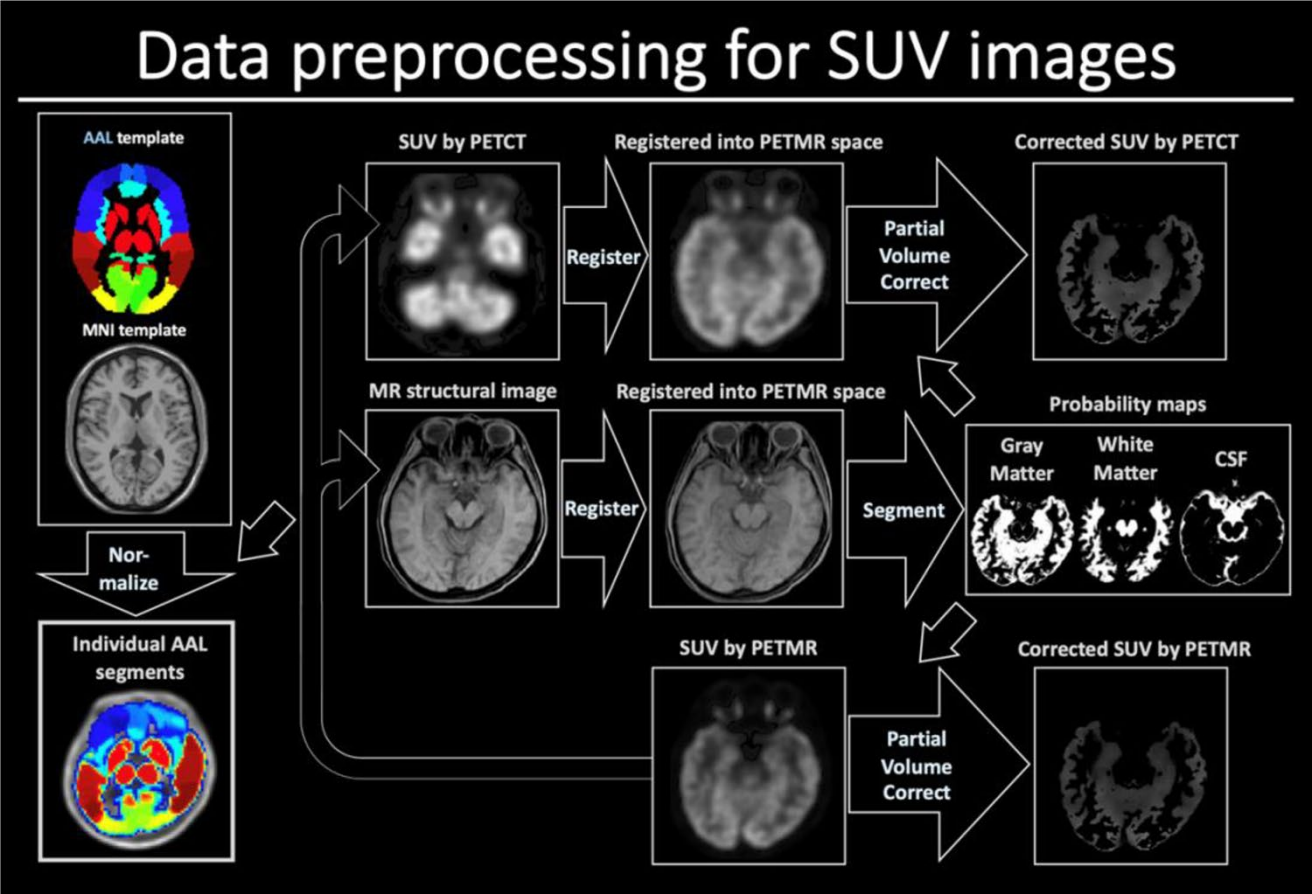
The image preprocessing for SUV images using PET/MR and PET/CT. All the individual PET/CT and structural images were registered into the orientation of individual PET/CT images. The structural images were then segmented into regions of gray matter (GM), white matter (WM), and CSF. The AAL3 template was normalized into the individual space. Then partial volume effect was corrected based on the segmented GM and WM maps for all the registered PET/CT and PET/MR images.

All data preprocessing steps were performed in the SPM12 software. First, the SUV of ^18^F-FBPA was calculated for each voxel of PET/CT, PET/MR ZTE-AC, and PET/MR AB-AC. Second, the SUV acquired by PET/CT and the structural LAVA images of each patient were co-registered into the spatial orientation of each SUV image dataset by PET/MR. Third, spatial segmentation was applied in the registered structural images for extraction of white matter, gray matter, and cerebrospinal fluid by normalizing the Montreal Neurological Institute space to each individual. Fourth, the partial-volume effect of all image data was corrected by the PETPVE12 toolbox. Fifth, to acquire precise tumor- and normal-tissue delineation, a senior physician determined the accurate delineation. Sixth, to evaluate the differences between different AC methods, the VOI-wise analysis was equipped with the Automated Anatomical Labeling template version 3 (AAL3)^23^ (Fig. 1).

Following the clinical usage, the core area of a tumor region was generated manually by volume delineation, which covered the whole-tumor contour in three dimensions and was exploited by applying a 50% SUV threshold. On the basis of the previous literature^10^, the SUVs of tumor regions were divided into SUVs of cerebellar lobule Crus I-II for the following T/N ratio with normal tissue is cerebellum analysis. In group-level analysis, the significant differences in T/N ratio levels between CT-AC, MRI ZTE-AC, and MRI AB-AC were evaluated by performing paired *t*-tests. Moreover, to measure and visualize the regional differences in SUVRs between PET/CT, PET/MR ZTE-AC, and PET/MR AB-AC, anatomical volumes of interest analyses by AAL3^23^ were performed to separate participants’ brains into eight major categories: the frontal region in light green, the limbic region in light blue, the occipital region in light yellow, the parietal region in light gray, the striatal region in. light orange, the temporal region in dark purple, the cerebellar region in dark gray, thalamic region in green and the anterior cingulate in yellow, (Fig. 3). To compare the gold standard PET/CT AC, Eq. (1) was applied to determine the comparisons of PET/MR AB-AC and PET/MR ZTE-AC with PET/CT AC individually. In the individual PET images of each patient, all manually-selected tumor regions were excluded on the basis of the MR images by an experienced physician who performed BNCT planning for > 12 years. Subsequently, the AAL3 template was applied to the residual brain regions to complete the normal-tissue delineation assessment. The VOIs in only ≤ 18 patients (< 2/3 of the enrolled subjects) were excluded in the following group analysis. Subsequently, to normalize the variation among patients, the SUVR was calculated from the SUV of each VOI divided into the average level of the SUV of the bilateral cerebellar lobule Crus I–II. In group-leveled data analysis, a paired *t*-test was also performed for the VOI-wise SUVR box plotting analyses between different AC approaches by PET/CT, PET/MR-ZTE, and PET/MR AB-AC (Fig. 4).

1 $${\text{The comparison of PET}}/{\text{MR AC and PET}}/{\text{CT }} = \;\frac{SUVRPETMR - SUVRPETCT}{{SUVRPETCT}}$$

### Ethical approval

This study was performed in line with the principles of the Declaration of Helsinki. Approval was granted by the Institutional Review Board of Taipei Veterans General Hospital (IRB: 2022-11-003AC).

### Consent to participate

The requirement for informed consent was waived due to the nature of retrospective study and was approved by the Institutional Review Board of Taipei Veterans General Hospital.

## Results

**Figure 2:**
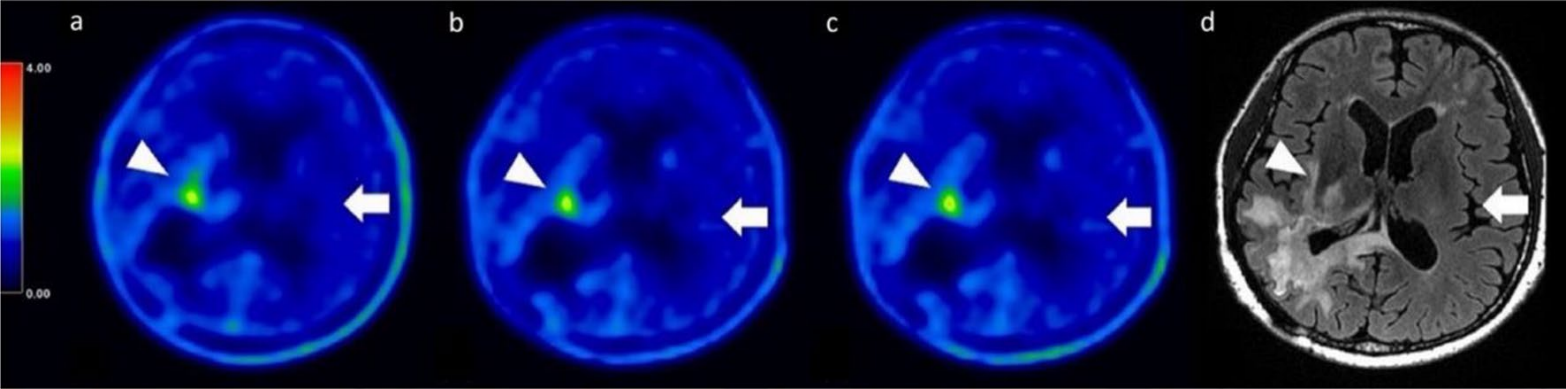
(**a**–**d**) Representative images of the 3 AC methods. While visual uptakes in target (tumor) lesion (arrowhead) and normal brain (arrow) showed no differences among PET/CT-AC (**a**), PET/MR ZTE-AC (**b**) and PET/MR AB-AC (**c**) in reference to the T2-Flair MRI (**d**), the subtle TNR however could be statistically discriminated.

**Figure 3:**
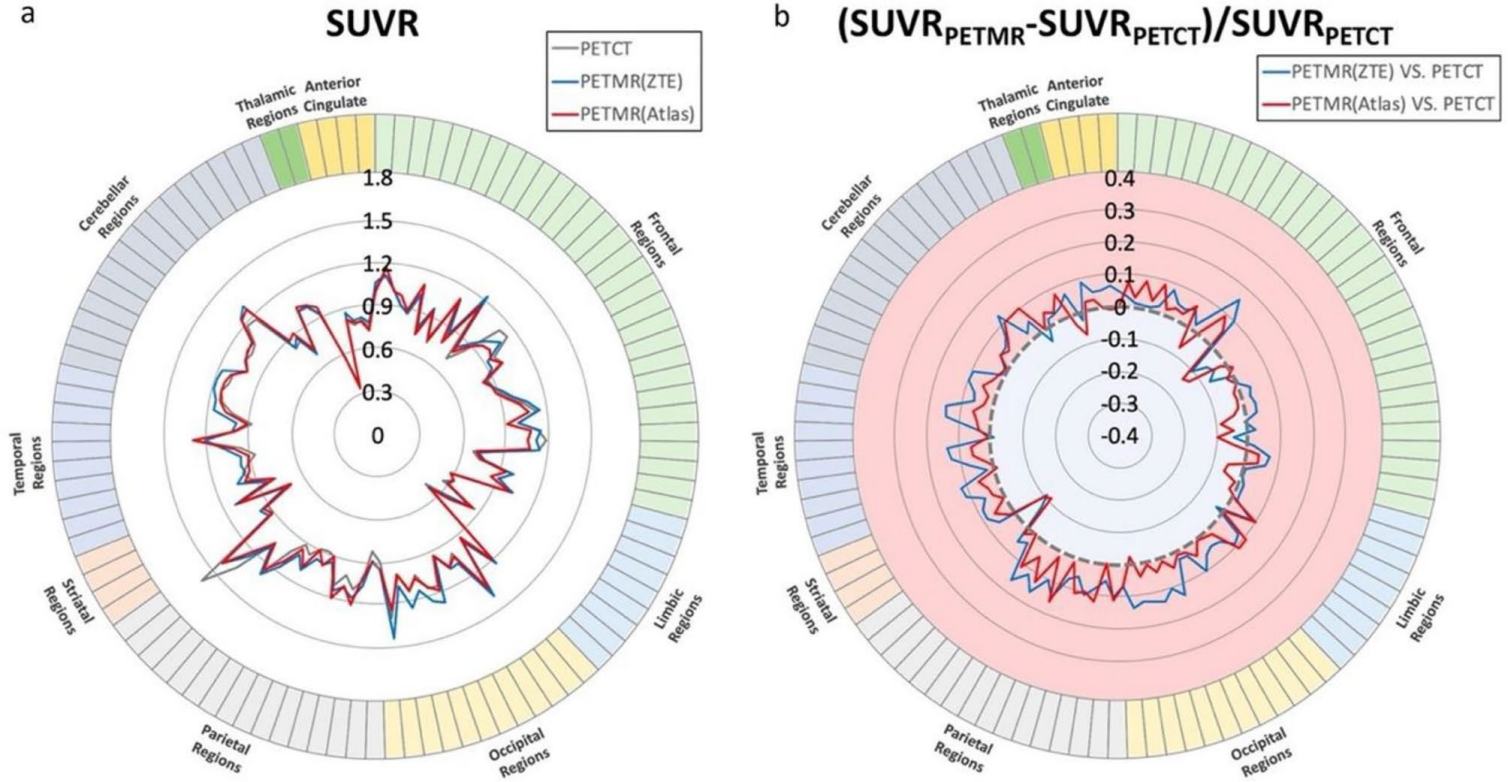
The averaged SUVR of normal brain ROIs and normal brain SUVR relative difference between PET/MR ZTE-AC and PET/MR AB-AC. The normal brain was separated into 166 sub-regions, those sub-regions contained less than 30 voxels were excluded in the following analysis. As compared with the SUVRs by PET/CT AC, almost all ROIs showed no significant difference of SUVRs by PET/MR ZTE-AC and PET/MRAB-AC (**a**) except the SUVR of right cerebellar lobule VIII by PET/MRZTE-AC that showed difference significantly (**b**).

The FBPA-PET image processing with CT-AC, MR ZTE-AC, or MR AB-AC showed no obvious differences in both tumor and normal brain uptakes by the visual inspection (Fig. 2a–d). Use of the AAL3 template showed that the whole brain could be separated into 166 sub regions, and those containing < 30 voxels were excluded in the following analysis. In the normal tissue determination using parametric methods, the differences in SUVRs between PET/CT and PET/MR AC methods ranged from − 15 to + 15%, and almost all VOIs showed no significant difference by visual inspection between SUVRs in CT-AC, ZTE-AC, and AB-AC (Fig. 3a). Only the SUVRs of the right cerebellar lobule VIII by PET/MR-ZTE were significantly higher than those by PET/CT (Bonferroni corrected *p* < 0.05) (Fig. 3b). To clarify the performance between two PET/MR AC methods, we normalized all the individual AC maps (with tumor regions removing) into MNI-template and acquire the group-levelled AC maps. The result showed that ZTE-AC shows less difference with the CT-AC than AB-AC (Fig. 5).

**Figure 4:**
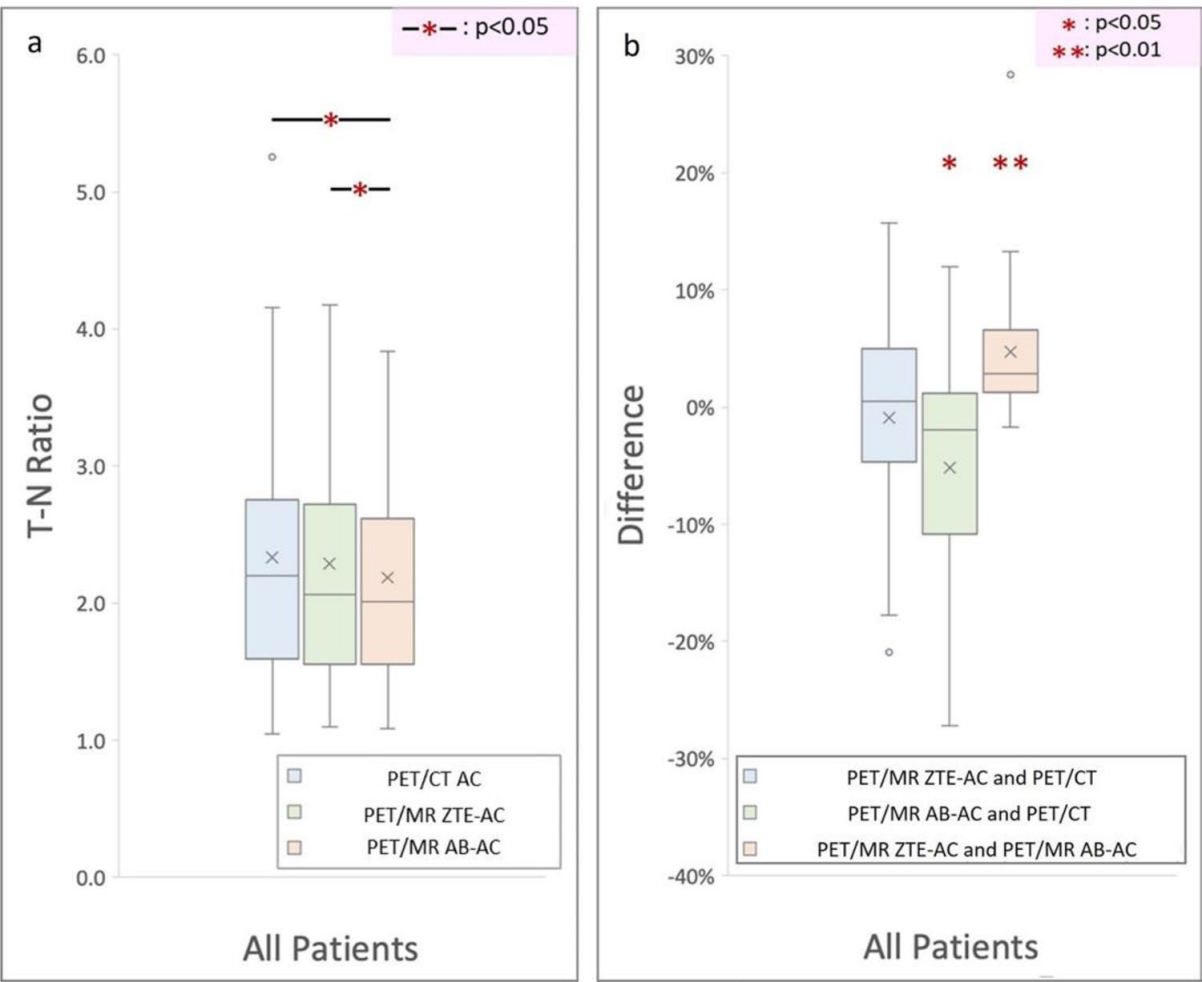
(**a**) Boxplot of the TNRs by PET/CT-AC, PET/MR ZTE-AC and PET/MR AB-AC. As compared to the TNRs by PETCT, those by PET/MR ZTE-AC showed no statistical significance (− 0.94 ± 8.97%; *p* = 0.58) but significantly lower values in those by PET/MR AB-AC (− 5.18 ± 9.52%; *p* < 0.05) (**b**).

In the T/N ratio assessment, the corrected ratios by PET/CT AC, ZTE-AC, and AB-AC were 2.34 ± 0.95, 2.29 ± 0.88, and 2.19 ± 0.80, respectively. Compared with the T/N ratios from PET/CT AC, those from ZTE-AC were not significantly different, whereas there were statistically significant differences between those for AB-AC and for CT-AC and ZTE-AC (Bonferroni corrected *p* < 0.05) (Fig. 4a). There were no significant differences in the T/N ratios between ZTE-AC and PET/CT AC, but the values were slightly lower (− 0.94% ± 8.97%; *p* = 0.58) than those of PET/CT AC. The T/N ratio was significantly lower for the AB-AC method (− 5.18% ± 9.52%; *p* < 0.05). The difference in the T/N ratios between ZTE-AC and AB-AC was also statistically significant (4.71% ± 5.80%; *p* < 0.01) (Fig. 4b).

## Discussion

Our data revealed that for [^18^F]-FBPA quantification in normal brain tissues, there were no significant differences in the T/N ratios among PET/MR, ZTE-AC, AB-AC, and the current-standard PET/CT-AC. In the brain tumors, the T/N ratios from PET/MR with ZTE-AC were similar to those for the current-standard PET/CT-AC, and significantly lower T/N ratios were observed for PET/MR AB-AC.

In the past decade, PET/CT has had an important role in the management of cancer patients because it not only offers accurate attenuation correction by low-energy CT, but also reduced the scanning time by > 40% relative to the time for whole-body scans by conventional PET study^24^. Moreover, in terms of whole-body scanning, PET/CT provides acceptable anatomic and morphologic information for diagnosis and treatment planning. However, for brain tumors, CT images from a PET/CT scanner render delineation of lesions and normal-brain tissues inferior to that from MRI images, so PET/CT images enable precise dosimetry planning before radiation therapy^25^.

The T/N ratio assessment of [^18^F]-FBPA-PET is crucial before BNCT to ensure suitable patient selection and treatment planning. However, in our clinical practice, we found some difficulties using PET/CT for measuring the T/N ratio in our brain tumor cohort, which was caused by determining tumor delineation, brain perifocal edematous effects, or anatomic structural changes following previous surgery or radiation therapy. Therefore, after the installation of a hybrid PET/MRI scanner, PET/MR gradually became the dominant modality of choice for patient evaluations before BNCT.

Although the anatomic and metabolic images from PET/MRI are favorable for BNCT evaluation, the reliability of PET/MR AC remains unclear. With the development of various MR AC methods, three prominent approaches, i.e., AB-AC, UTE-AC, and ZTE-AC, have been frequently adopted. The first PET/MR AC method is AB-AC. To generate the AB-AC, the segmentation-driven AC maps are classified by Dixon-based MR images and divided into different tissues, such as air, bone, and soft tissue, with standardized attenuation coefficient values^26^. Then, the AB-AC maps can be equipped by combining template and segmentation methods, including CT images, MRI models, and a brain mask^27–30^. The second method is UTE-AC (Biograph mMR PET/MRI from Siemens Healthcare), which utilizes the short T_2_^*^ to acquire tissue signals as the attenuation map (μ-map). This method has established a linear relationship between the relaxation rate, *R*_2_^*^, stemming from 1/T_2_^*^ and CT density which comes from the attenuation values in bone MRI images^31,32^. Currently, the ZTE-AC (SIGNA PET/MRI from GE Healthcare) method is based on the pseudo-CT attenuating property to develop the subject-specific AC map^17^. The ZTE-AC approach enables precise quantitative evaluation of brain PET according to previous FDG studies^18,21, 22^. BNCT is a type of α-particle radiotherapy. Precise delineation of the tumor lesion and separating it from normal tissue based on [^18^F]-FBPA PET result is crucial for successful treatment and minimization of side effects. However, there have been no reports that focused on optimizing [^18^F]-FBPA brain PET/MR imaging for the same purpose. The rationale for this study was to observe the imaging outcomes after applying different attenuation-correction methods, i.e., ZTE-AC and AB-AC, for [^18^F]-FBPA PET/MR quantification accuracy and compare them with the current benchmark of PET/CTAC.

In the present study, we could not visually determine the differences between [^18^F]-FBPA PET images from CT-AC, MR ZTE-AC, or MR AB-AC (Fig. 2a–d). To reveal the precise uptake differences in normal brain tissues, we used AAL3 segmentation to perform automated labeling with subtle brain segmentations and to calculate the proportion of labeled brain sub-regions^23^. Small SUVR differences in all normal brain regions between the three attenuation-correction methods were observed, but only higher SUVR values in the right cerebellar lobule III were observed for ZTE-AC (Fig. 3a,b). Some regions, such as the thalamus and anterior cingulate, exhibited high agreement between CT-AC, MR ZTE-AC, and MR AB-AC.

Our study found no significant differences in the [^18^F]-FBPA T/N ratio between the current gold standard CT-AC and ZTE-AC, whereas a significantly lower T/N ratio of AB-AC (Fig. 4a,b) reflects a problem of underestimation. These results suggested that PET/MRI AB-AC may underestimate the [^18^F]-FBPA T/N ratio and could result in the failure of patient recruitment in the BNCT treatment management. The main reason for underestimating AB-AC in the T/N ratio may be generated by undervaluing the tumoral uptake relative to that of ZTE-AC and CT-AC. If ZTE-AC is used, the T/N ratio in [^18^F]-FBPA PET data might be closer to that in real-world settings that perform CT-AC by generating a pseudo-CT. This pseudo-CT not only provides a linear correlation with CT density of the bone in Hounsfield units (HU) but also a subject-specific AC map that provides flexibility and an advanced AC calculation^17^. Moreover, ZTE intensity in some soft-tissue regions with misclassification between air and bone, such as spongious temporal bones, frontal sinus region, mastoid region, and nasal-sinus cartilage, is closer to the phenomenon of the partial-volume effect in the same regions observed by PET/CT^17,21^. In the brain region, ZTE-AC uses classification along with the CT information in the (0,100) HU soft-tissue range^21^. Although previous studies have provided significant differences that may be seen in the neck that are related to the different procedures used in PET/CT and PET/MR^17^, all subjects recruited in this study were examined for brain tumors. Till date, no literature has discussed the effect of ZTE-AC and AB-AC on [^18^F]-FBPA PET/MRI. Our findings suggest that PET/MR imaging using ZTE-AC can provide [^18^F]-FBPA-PET quantification superior to that of AB-AC because the statistical results from PET/MR ZTE-AC are more similar to those in PET/CT AC. Using ZTE-AC on [^18^F]-FBPA, PET/MRI may have a crucial role in BNCT pretreatment planning in determining the T/N ratio.

Regarding the clinical impact, 12 patients can be included in BNCT treatments using PET/CT AC measurements. Sixteen patients cannot be included in because the standard of T/N ratio ≥ 2.5 was adopted. Similar results were observed with ZTE-AC PET/MRI, when T/N ratio ≥ 2.5 was found in 13 patients. However, only 9 subjects received T/N ≥ 2.5 using AB-AC PET/MRI. Three patients will be excluded from BNCT treatments attributed to the underestimation of T/N from AB-AC. Consequently, 25% of subjects is eligible and can be include in BNCT treatments by using PET/MR ZTE-AC and PET/CT AC.

Our study had some limitations that should be considered. First, the different spatial resolutions between two hybrid machines should be considered carefully. Another limitation is the small study sample size. However, a larger-scale prospective study might be difficult to conduct because (^18^F)-FBPA-PET and the subsequent BNCT therapy are part of a compassionate treatment combination with strict regulation from our institutional review board. We hope a further multi-center meta-analysis focusing on (^18^F)-FBPA-PET using hybrid PET/MRI would be able to validate our results. A third study limitation is that because the principles of ZTE are related to the use of pseudo-CT for AC, some uncertain factors might affect the ZTE-AC map. For example, in our study, we found two outliers shown in Fig. 4b. One patient was affected by a large pseudo-skull material following his previous surgery. This unknown material probably led to misleading characteristics of the material on the reconstruction and generated AC errors in ZTE-AC^33^. Finally, we observed a recurrent tumor sticking to the meninges and scalp, which may cause tumor delineation difficulties and AC errors. Further studies are warranted to better evaluate possible confounding factors in [^18^F]-FBPA-PET treatment planning using PET/MR.

**Figure 5:**
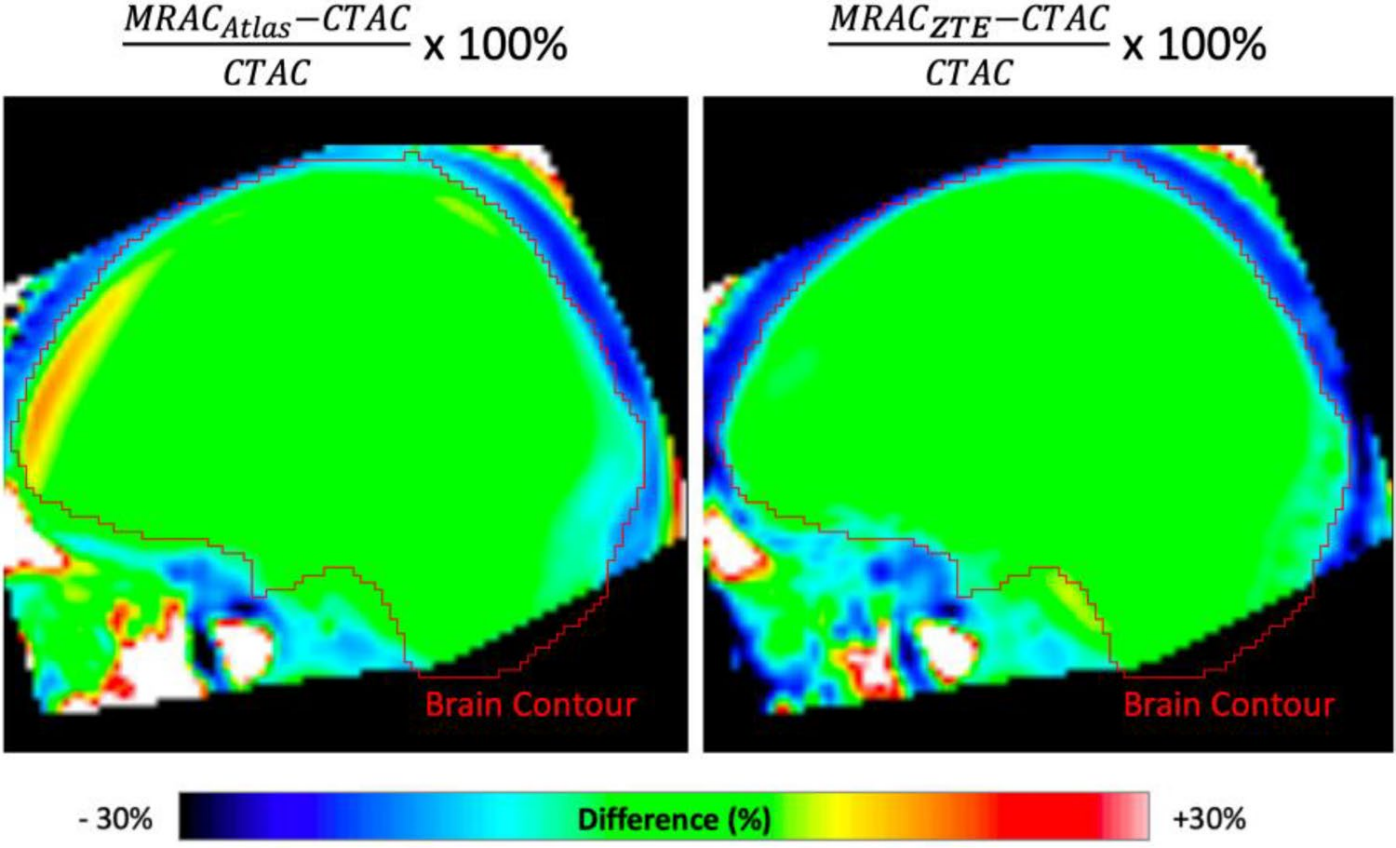
The difference between two PET/MR-ACs and CT-AC of normal tissues. To clarify the performance between two PET/MR AC methods, we normalized all the individual AC maps (with tumor regions removing) into MNI-template and acquire the group-levelled AC maps. The difference of normal tissues is apparently less between PET/MR ZTE-AC and CT-AC than it between PET/MR AB-AC and CT-AC.

## Conclusion

These findings suggest that the routinely quantitative use of ZTE-AC on [^18^F]-FBPA-PET/MR for BNCT therapeutic planning of brain tumors might provide a closer result to the standard PET/CT AC.

## Data Availability

The datasets generated and/or analyzed during the current study are available from the corresponding author on reasonable request.
